# Reinforcement learning in medical image analysis: Concepts, applications, challenges, and future directions

**DOI:** 10.1002/acm2.13898

**Published:** 2023-01-10

**Authors:** Mingzhe Hu, Jiahan Zhang, Luke Matkovic, Tian Liu, Xiaofeng Yang

**Affiliations:** ^1^ Department of Radiation Oncology School of Medicine Emory University Atlanta Georgia USA; ^2^ Department of Computer Science and Informatics Emory University Atlanta Georgia USA

**Keywords:** reinforcement learning

## Abstract

**Motivation:**

Medical image analysis involves a series of tasks used to assist physicians in qualitative and quantitative analyses of lesions or anatomical structures which can significantly improve the accuracy and reliability of medical diagnoses and prognoses. Traditionally, these tedious tasks were finished by experienced physicians or medical physicists and were marred with two major problems, low efficiency and bias.

In the past decade, many machine learning methods have been applied to accelerate and automate the image analysis process. Compared to the enormous deployments of supervised and unsupervised learning models, attempts to use reinforcement learning in medical image analysis are still scarce. We hope that this review article could serve as the stepping stone for related research in the future.

**Significance:**

We found that although reinforcement learning has gradually gained momentum in recent years, many researchers in the medical analysis field still find it hard to understand and deploy in clinical settings. One possible cause is a lack of well‐organized review articles intended for readers without professional computer science backgrounds. Rather than to provide a comprehensive list of all reinforcement learning models applied in medical image analysis, the aim of this review is to help the readers formulate and solve their medical image analysis research through the lens of reinforcement learning.

**Approach & Results:**

We selected published articles from Google Scholar and PubMed. Considering the scarcity of related articles, we also included some outstanding newest preprints. The papers were carefully reviewed and categorized according to the type of image analysis task. In this article, we first reviewed the basic concepts and popular models of reinforcement learning. Then, we explored the applications of reinforcement learning models in medical image analysis. Finally, we concluded the article by discussing the reviewed reinforcement learning approaches’ limitations and possible future improvements.

## 1 INTRODUCTION

**FIGURE 1:**
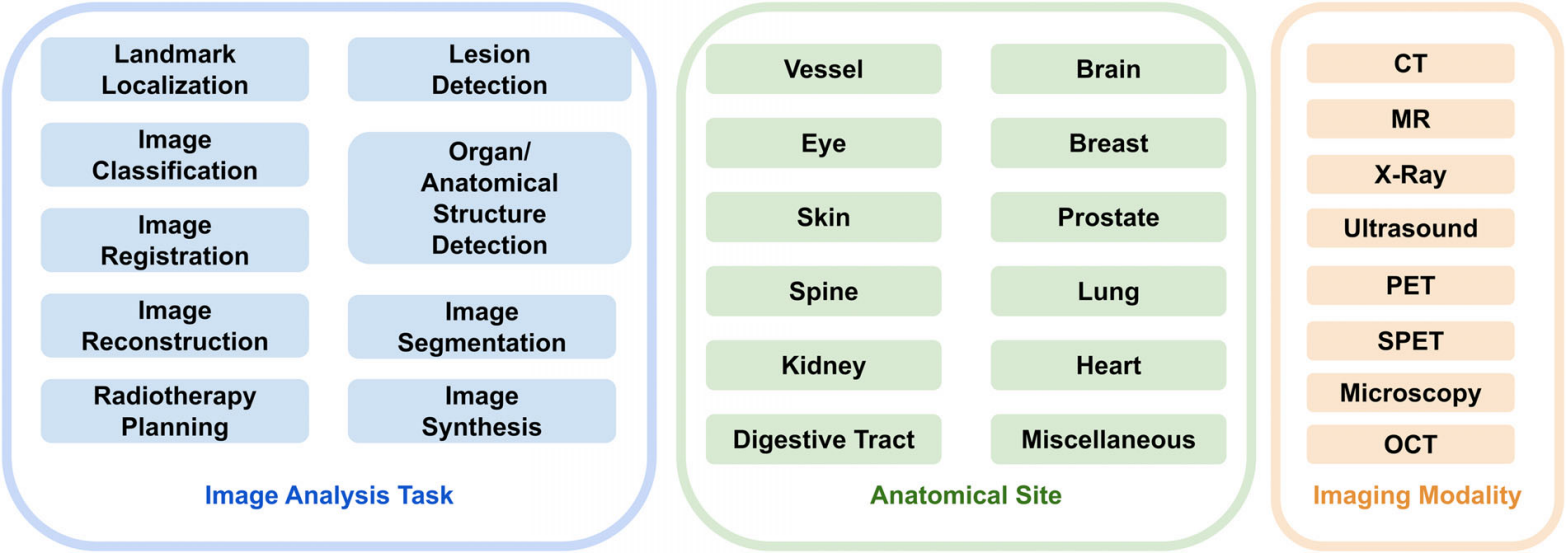
Range of our review article. Blue box: covered image analysis tasks; green box: covered anatomical sites; yellow box: covered imaging modalities

The purpose of medical image analysis is to mine and analyze valuable information from medical images by using digital image processing to assist physicians in making more accurate and reliable diagnoses and prognoses. Common imaging modalities include computed tomography (CT), magnetic resonance imaging (MRI), ultrasound, single photon emission computed tomography (SPECT), positron emission tomography (PET), X‐ray, optical coherence tomography (OCT), and microscope. Medical image processing can also be classified according to specific processing tasks, which typically include classification, segmentation, registration, and recognition. Figure 1 shows the range of our review article.

Improvements in imaging technology and equipment have reduced imaging time and improved image resolution. At the same time, the size of medical images has experienced an unprecedented surge with the trend of high dimensionality. As such, the traditional manual analysis of medical images by physicians has become tedious and inefficient. Partnered with engineers, physicians moved to automate the process through machine learning. Many excellent algorithms in the field of natural image analysis have also shown good results in the field of medical imaging.^1^

Reinforcement learning (RL) is neither supervised nor unsupervised learning. The goal of reinforcement learning is to achieve the maximum expected cumulative reward.^2^

**FIGURE 2:**
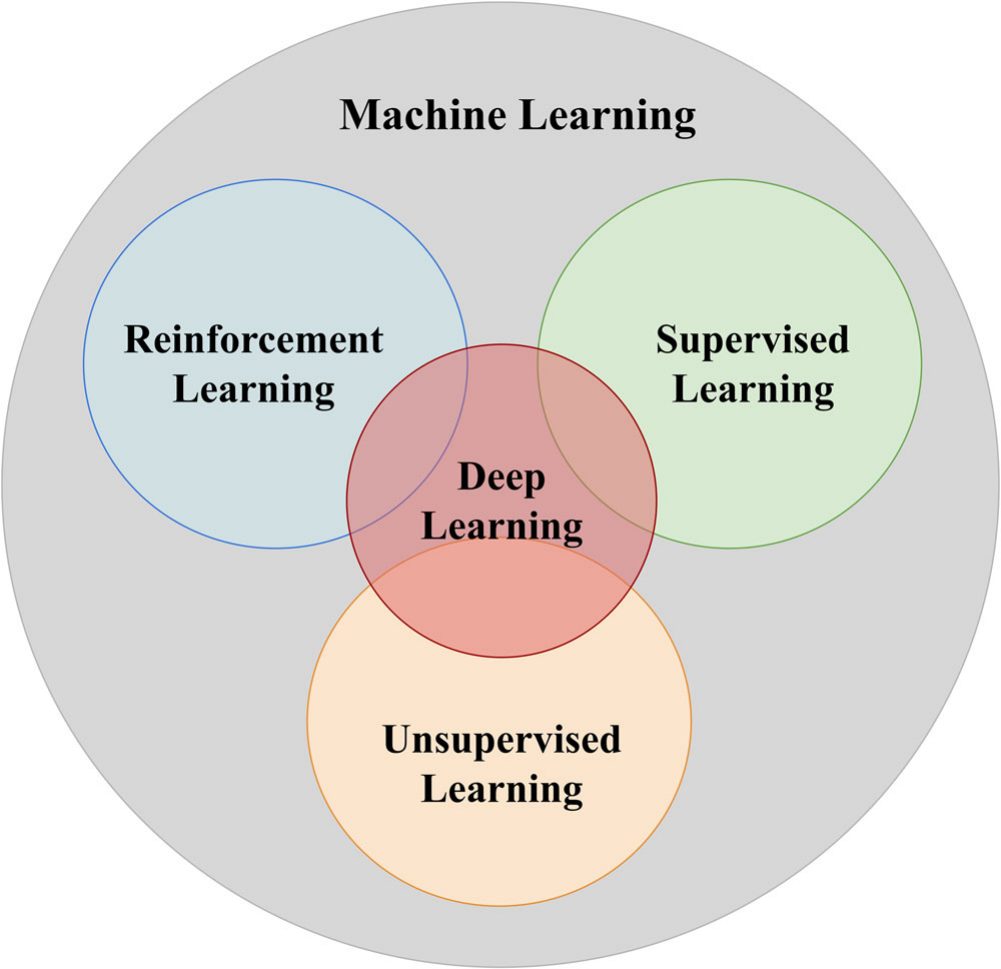
Relationship between machine learning, supervised learning, unsupervised learning and reinforcement learning

Figure 2 shows the relationship between machine learning, supervised learning, unsupervised learning, reinforcement learning, and deep learning.

**FIGURE 3:**
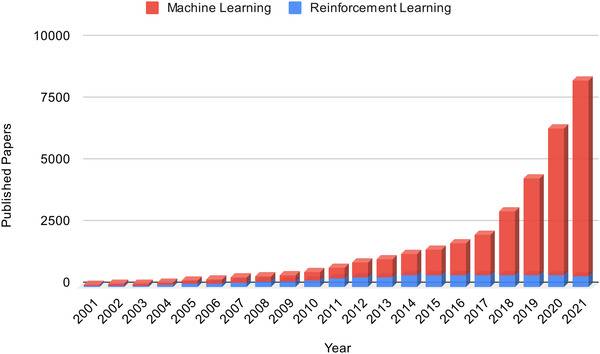
Trends of number of published machine learning papers and reinforcement learning papers in medical image analysis. This figure is made by separately searching the keywords “Machine Learning AND (Medical Imaging OR (Medical Image Analysis))” and “Reinforcement learning AND (Medical Imaging OR (Medical Image Analysis))” in PubMed. The number of papers published each year is counted

The number of published RL‐related papers has grown rapidly in the past two decades. State‐of‐the‐art RL models have been applied to solve problems that are difficult or infeasible with other machine learning approaches, such as playing video games,^3^, ^4^, ^5^ natural language processing,^6^ and autonomous driving.^7^ These RL methods have achieved outstanding performances. However, attempts to exploit the technical developments in RL in the medical analysis field are scarce. Figure 3 shows the trends of number of published machine learning and reinforcement learning papers in medical image analysis. Despite the growth, the number of published RL papers still only constitutes a small subset of machine learning in medical image analysis. On the other hand, RL methods have unique advantages in dealing with medical image data, including:

- RL models do not require a massive amount of data annotation. It can learn to achieve the final goal by interacting with the environment and exploiting past experience. RL models are less biased since they won't inherit bias from the labels made by human annotators.
- RL agents can learn from sequential data through a goal‐oriented process. In addition to developing experience from known data, it can also explore new solutions. RL can even surpass human experts when solving the same problem.

The review article is based on Synthesis Methodology.^8^

**FIGURE 4:**
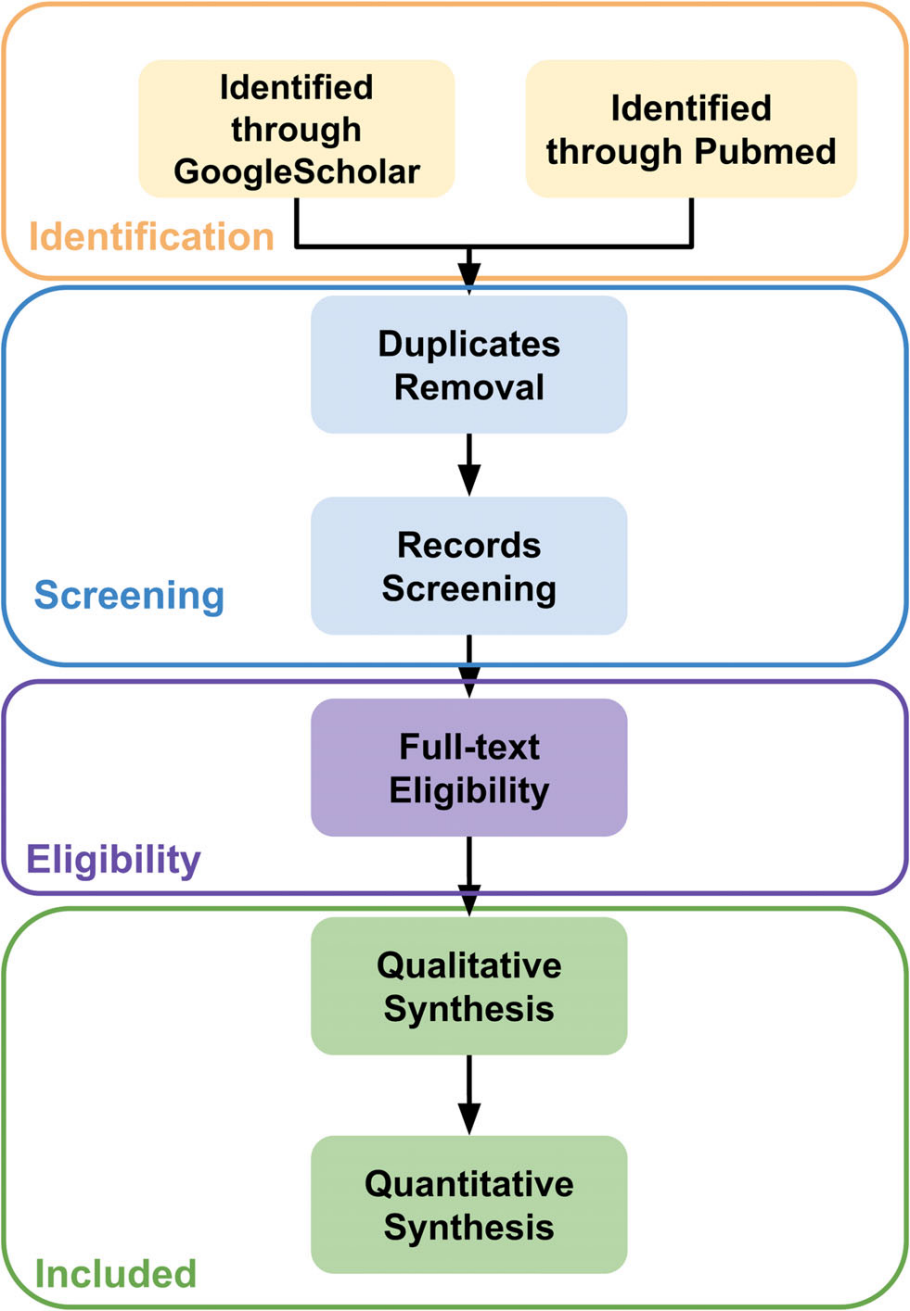
Flows of information through the different phases of a systematic review

The methodology Preferred Reporting Items for Systematic Reviews and Meta‐Analyses (PRISMA) was followed.^9^ First, the following pattern was searched in Google Scholar and PubMed: Clustering AND (Medical OR CT OR MR OR Ultrasound OR X‐ray OR OCT) AND IMAGE AND Segmentation. Then, the duplicate papers were removed. We set the qualified publication date to 2010. The remaining papers went through qualitative synthesis and quantitative synthesis. The summary of the selection process is shown in Figure 4.

**FIGURE 5:**
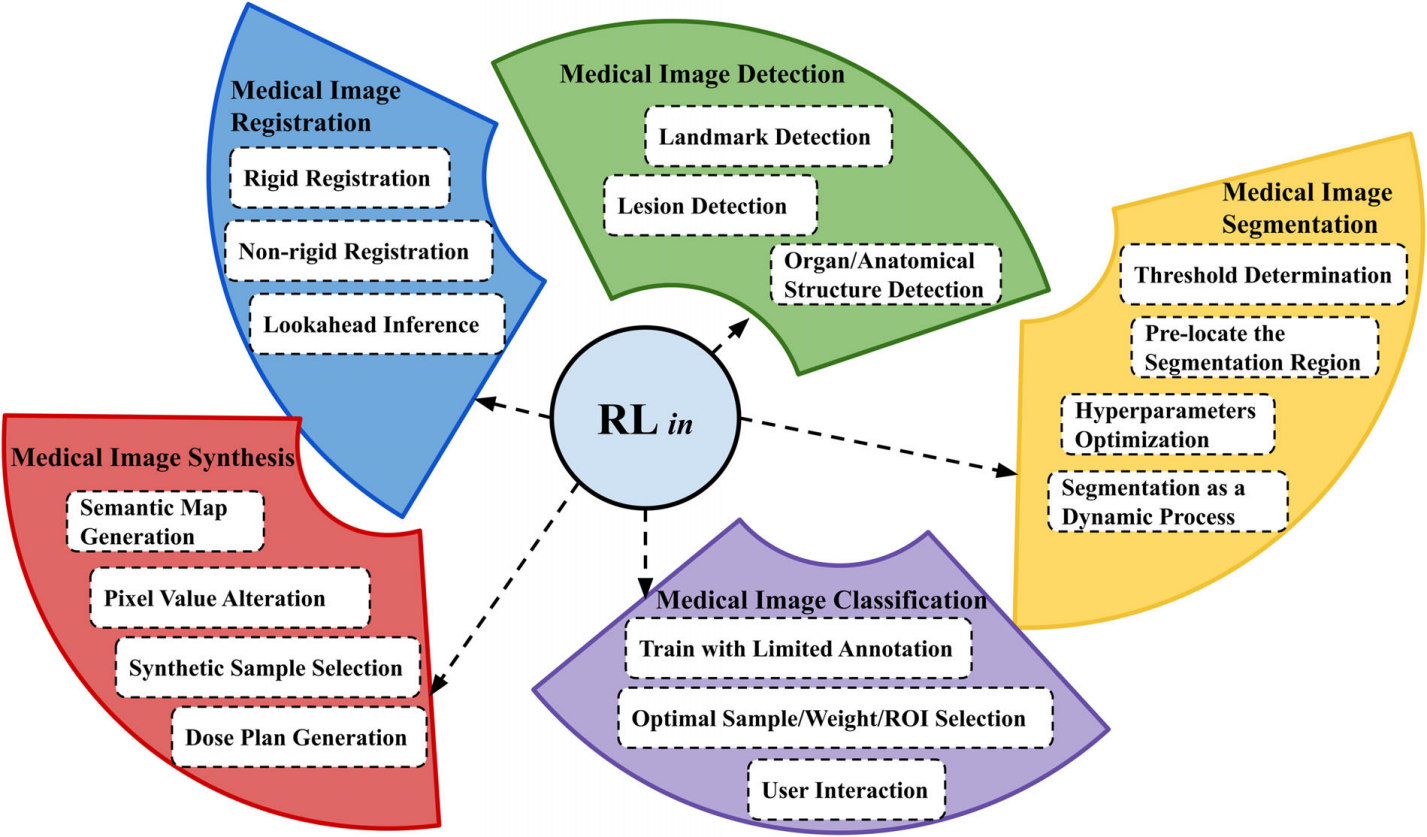
Overview of contents in the application section

By reviewing content, analyzing common points, and comparing differences between these papers, we aim to enable our readers to have a better understanding of RL so that they can formulate and solve their medical image analysis research through the lens of RL. For the next two sections, we first prepared the readers with basic knowledge of RL. Then, we showed how to apply RL in different medical image analysis tasks. This section will review the state‐of‐the‐art works applying RL in medical image analysis. Figure 5 shows a preview of the main contents of the application section. Readers who are familiar with RL algorithms can directly go to the application section.

## 2 REINFORCEMENT LEARNING BASICS

In this subsection, we provided a list of terminologies that frequently appear in RL papers. Some terminologies may appear in definitions of other terminologies before they are defined.

**FIGURE 6:**
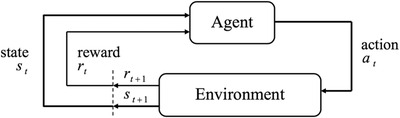
Agent Environment Interaction (Key components of RL models). Adapted from Rafati & Noelle.^10^

- *Action (A)*: The way that an agent interacts with the environment and includes all possible actions that an agent can perform.
- *Agent*: The model we attempt to build that interacts with the environment and takes actions.
- *Environment*: The content that the agent is interacting with. While providing feedback after the agent takes action, the environment itself is also changing.
- *State (S)*: A frame of an environment and includes all states that an agent will go through.
- *Reward (R)*: A value that the environment assigns to the agent after an action. A positive reward means an increased probability of achieving the goal, while a negative reward means a decreased probability. *R* includes all possible reward values the environment may feed back to the agent.
- *Episode*: The collection of states that an agent goes through, from the initial state to the terminal state.
- *Transition probability* $P(s^{\prime }|s,a)$: The probability of transiting to state$\;s^{\prime }$ to from the current state *s* by taking the action *a*.
- *Policy* π(a|s): Instructs the agent to choose among actions A under the current state *s*.
- *Return (G)*: The cumulative discounted future reward.
- $G_{t}=r_{t}+\gamma r_{t+1}+\gamma^{2}r_{t+2}$, where t is the time and γ is the discount factor.
- *State value* $V^{\pi }\left(s\right)$: The expected amount of return from the current state.
- $V^{\pi }\left(s\right)=E\left[G_{t}|s_{t}=s\right]$, where E is the expectation.
- *Action value* $Q^{\pi }\left(s,a\right)$ *(Q value)*: The expected amount of return from the current state, taking action s. $Q^{\pi }\left(s,a\right)=E\left[G_{t}|s_{t}=s,a_{t}=a\right]$
- *Optimal action value*: $Q^{★}\left(s,a\right)$: $Q^{★}\left(s,a\right):Q^{★}\left(s,a\right)=\max_{\pi }Q^{\pi }\left(s_{t},a_{t}\right)$
- *Agent environment interaction*: Figure 6 shows how the agent interacts with the environment.

Numerous algorithms have been created through the development of RL theory. Benefiting from its combination with deep learning, RL is now capable of handling progressively more complex scenarios in modern applications. Regardless of how complex these state‐of‐the‐art algorithms are, they can be mainly divided into two categories: model‐ based RL and model‐free RL. As its name indicates, model‐based RL attempts to explain the environment and create a model to simulate it. Model‐free RL, however, will only update its policy by interacting with the environment and observing the rewards.

**FIGURE 7:**
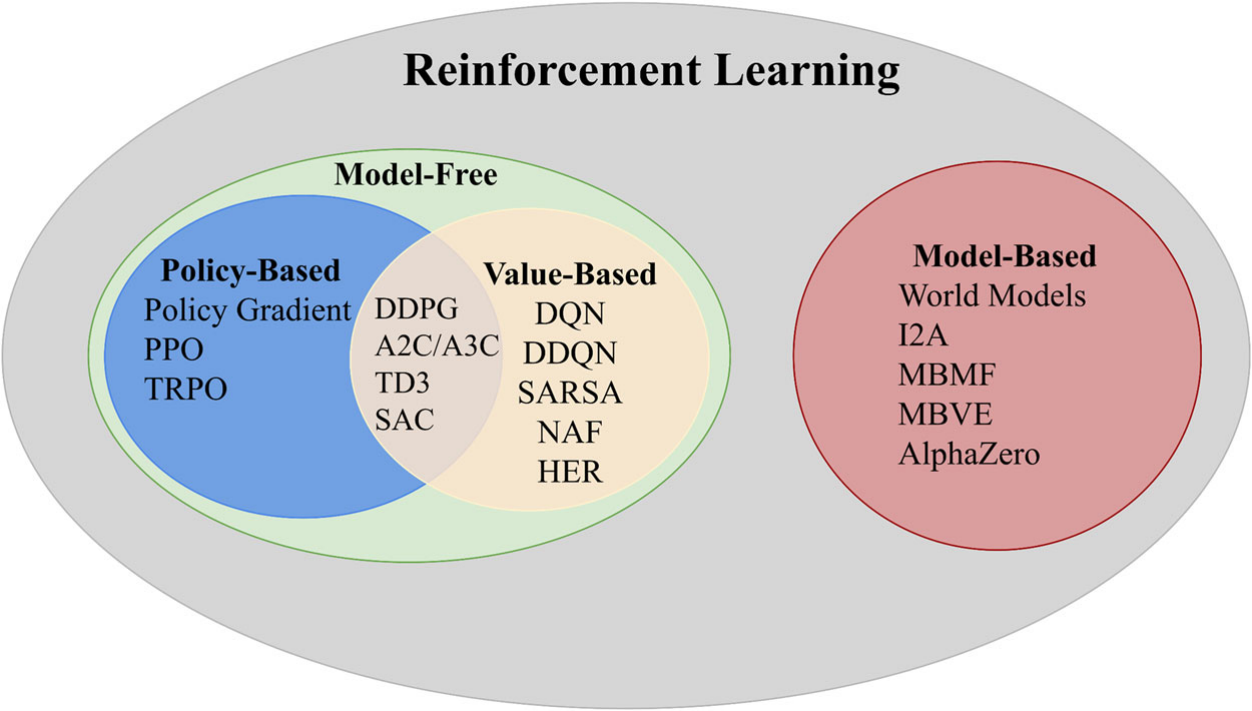
Reinforcement learning algorithms taxonomy

We can further divide the model‐free RLs into value‐based and policy‐based according to whether the algorithm is optimizing the value or policy function. Value‐based RLs are widely applied for discrete action space problems, while policy‐based RLs are suitable for both discrete and continuous action spaces. Some RL algorithms are based on both value and policy, like DDPG,^11^ TD3^12^ and SAC.^13^ Figure 7 shows the taxonomy of popular RL algorithms. In our review, all the RL algorithms were model‐free, and the most popular ones were DQN, DDQN, A2C, and DDPG. Below, we included brief introductions of these popular RL algorithms commonly used in medical image analysis.

### 2.1 DQN

**FIGURE 8:**
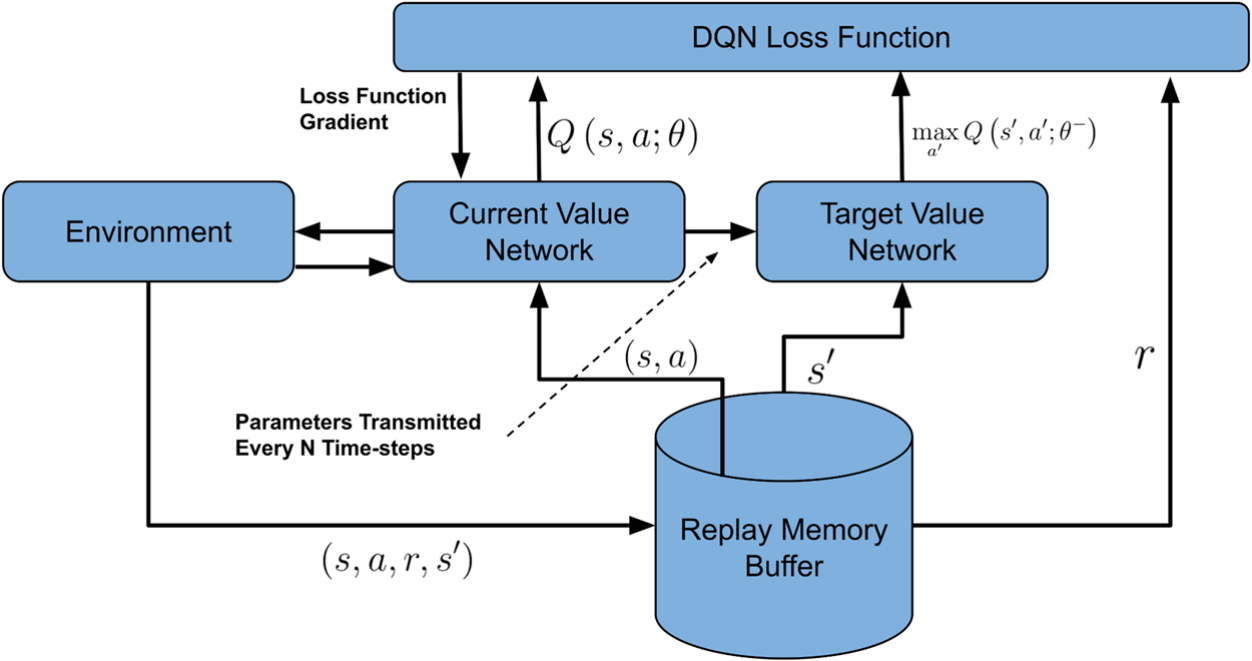
Workflow of DQN algorithm

The Deep Q‐Network (DQN) was first proposed^3^, ^4^ to solve some complex computer perception vision problems. It combined the idea of the traditional Q‐learning method^14^ and the deep CNN.^15^ The main motivation of DQN was to solve the problem where the Q‐table can only store a limited number of states, while in real‐life scenarios, there could be an immense or even infinite number of states. DQN adopted the experience replay mechanism that randomly sampled a small batch of tuples from the replay buffers during the training process. The correlations between the samples were significantly reduced, which led to better algorithm robustness. Another improvement over Q‐learning was that DQN used a deep CNN to represent the current Q‐function and used another network to define the target Q value. The introduction of the target Q value network reduced the correlation between the current and target Q values. Figure 8 shows the workflow of the DQN.

### 2.2 DDQN

DQN is one of the most popular RL algorithms currently applied in medical image analysis. The optimization target in DQN is represented as $r+\gamma \max_{a^{\prime }}Q\left(s^{\prime },a^{\prime }|\theta_{i}^{-}\right)$. The selection and evaluation of actions are all based on the network's same parameter. The action is simply chosen according to the maximum Q value, which may lead to overestimation of the Q value. The Double DQN (DDQN)^16^ used two separate networks for selection and evaluation. Here, the target Q value was written as $r+\gamma Q(s^{\prime },\mathrm{argma}x_{a}Q\left(s^{\prime },a|\theta_{i}\right),\theta_{i}^{-})$, which achieved a more stable learned policy than DQN.

### 2.3 Actor critic

The Actor‐Critic (AC)^17^ algorithm mixed the idea of policy gradient and Time Difference (TD) learning and could handle continuous action space problems and update the policy in an efficient, stepwise manner. The actor was the policy function $\pi_{\theta }\left(a|s\right)$ that learned the policy using gradient descent to achieve the highest possible reward and the critic was the value function $V^{\pi }\left(s\right)$ that used the TD error to assess the current policy.

### 2.4 A2C/A3C

A2C^18^ and A3C^19^ are improved versions of the vanilla AC algorithm that introduced the parallel architecture. Each agent included a global network and multiple workers that ran independently. Every worker gathered different experiences and calculated different gradients. A2C worked in a synchronous way and A3C worked in an asynchronous way tackling the different parameters and gradients. In this context, synchronous means that different workers share the same policy and the time to update the policy is the same, which leads to A2C typically converging faster than A3C.

### 2.5 DDPG

DDPG is a type of AC‐based algorithm but learns the off‐policy. Like DQN, samples generated by the random policy are stored in the memory replay buffer. However, DQN can only solve control problems with discrete actions, while DDPG can solve the problems in the continuous action space and shows excellent efficiency in finding the optimal policy. However, for some random environments, such as low signal‐to‐noise‐ratio images, the deterministic policy gradient strategy adopted by DDPG is not suitable.

## 3 RL IN MEDICAL IMAGE ANALYSIS

### 3.1 Medical image detection

#### 3.1.1 Overview of works

##### Landmark detection

**FIGURE 9:**
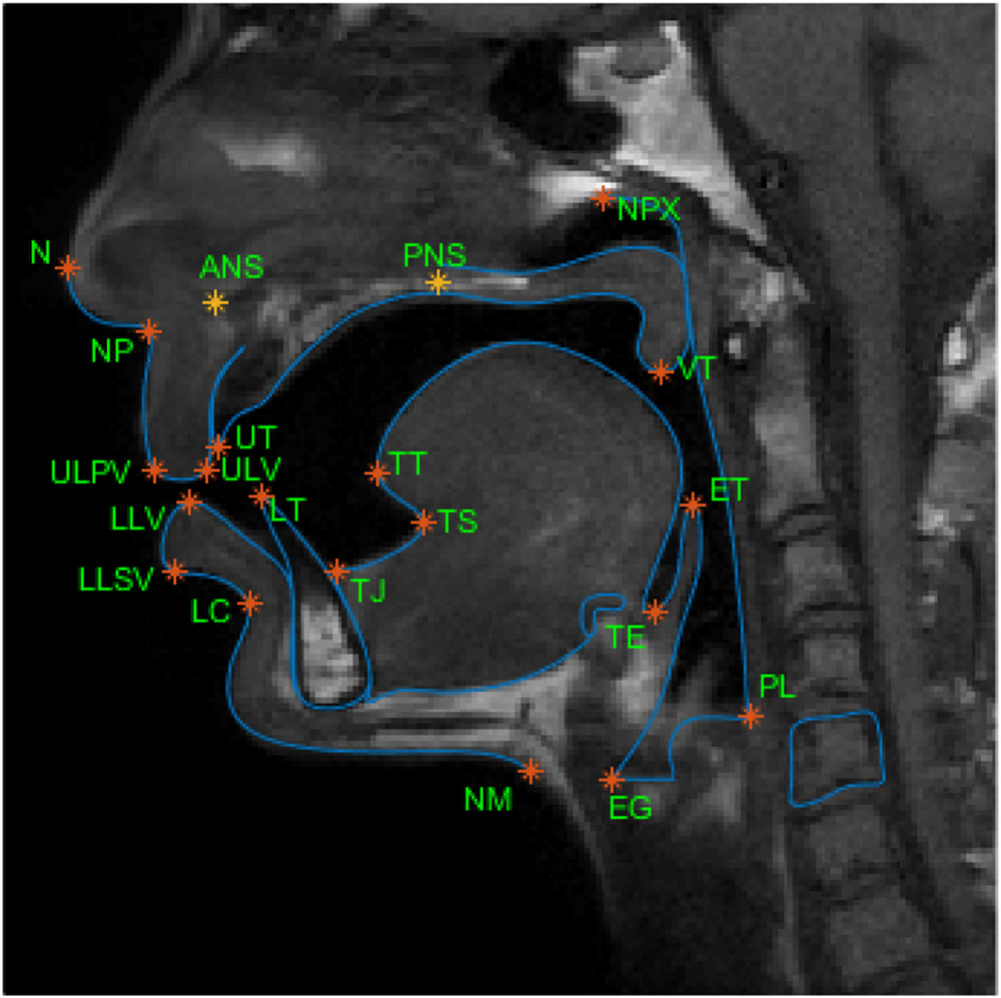
Vocal tract landmarks from MRI image Courtesy of Eslami et al.^82^

Anatomical landmarks are biological coordinates that can be reallocated repeatedly and precisely on images produced by different imaging modalities, such as CT, ultrasound, and MRI. The accurate detection of anatomical landmarks is the basis for further medical image analysis tasks. Figure 9 is an example of vocal tract landmarks from an MRI image.

Many automatic algorithms for anatomical landmark detection predate the use of RL models. However, landmark detection, especially 3D landmark detection, can be challenging and cause the failure of these algorithms.^20^ Moreover, the computation of features and hyperparameter selection of the system may not be optimal given the involvement of human decisions. The researchers attempted a different paradigm to address this problem, notably to translate the landmark detection tasks to reinforcement learning problems, which was the common goal of the papers we reviewed. The most essential and intricate task in these papers is designing the state space, action space, and reward space before training the models.

**FIGURE 10:**
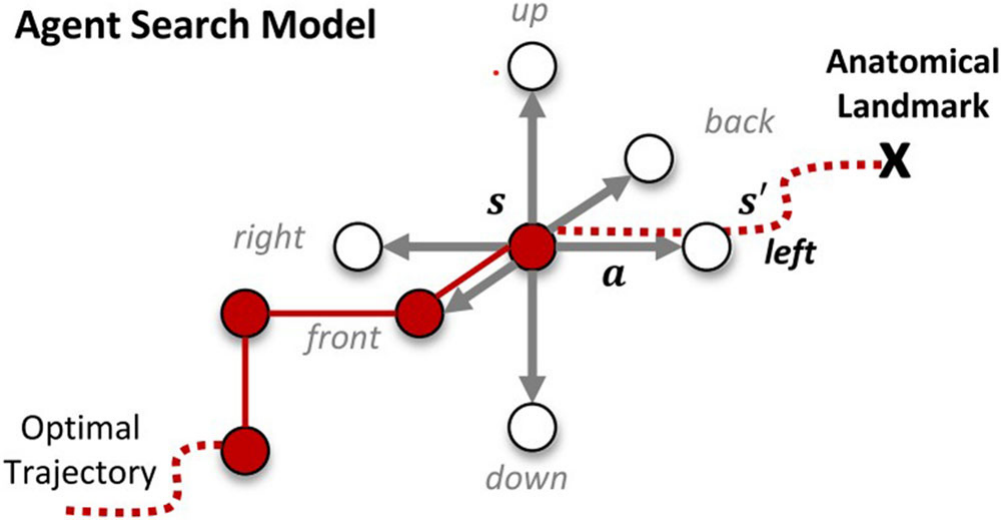
Possible actions of a 3D landmarks detection task. Courtesy of`Ghesu et al.^20^

Ghesu et al.^21^ was one of the first papers that attempted to use RL for anatomical landmark detection. In an image I, $\vec{p_{GT}}$ denoted the location of anatomical landmark, and $\vec{p_{t}}$ denoted the location at the current time. State space S was the collection of all possible states $s_{t}=I\left(\vec{p_{t}}\right)$. Action space A was the collection of all possible actions by which the agent can move to the adjacent position, as illustrated by Figure 10. Reward space R was defined as $||\vec{p_{t}}-\vec{p_{GT}}|{|}_{2}^{2}-{\left||\vec{p_{t+1}}-\vec{p_{GT}}|\right|}_{2}^{2}$ which impelled the agent to move closer to the target anatomical landmark. A deep learning model was applied to approximate the state value function. The parameters were updated according to gradient descent, and the error function was:

(1) $$\begin{array}{c} \hat{\theta_{i}}=\arg\min_{\theta_{i}}E_{s,a,r,s^{\prime }}\left[{\left(y-Q\left(s,a;\theta_{i}\right)\right)}^{2}\right]+E_{s,a,r}\left[V_{s^{\prime }}\left(y\right)\right] \end{array}$$

This deep Q‐learning‐based method beat the existing top systems not only in accuracy but also in speed. This design of action, state, and reward spaces became a standard method.

**FIGURE 11:**
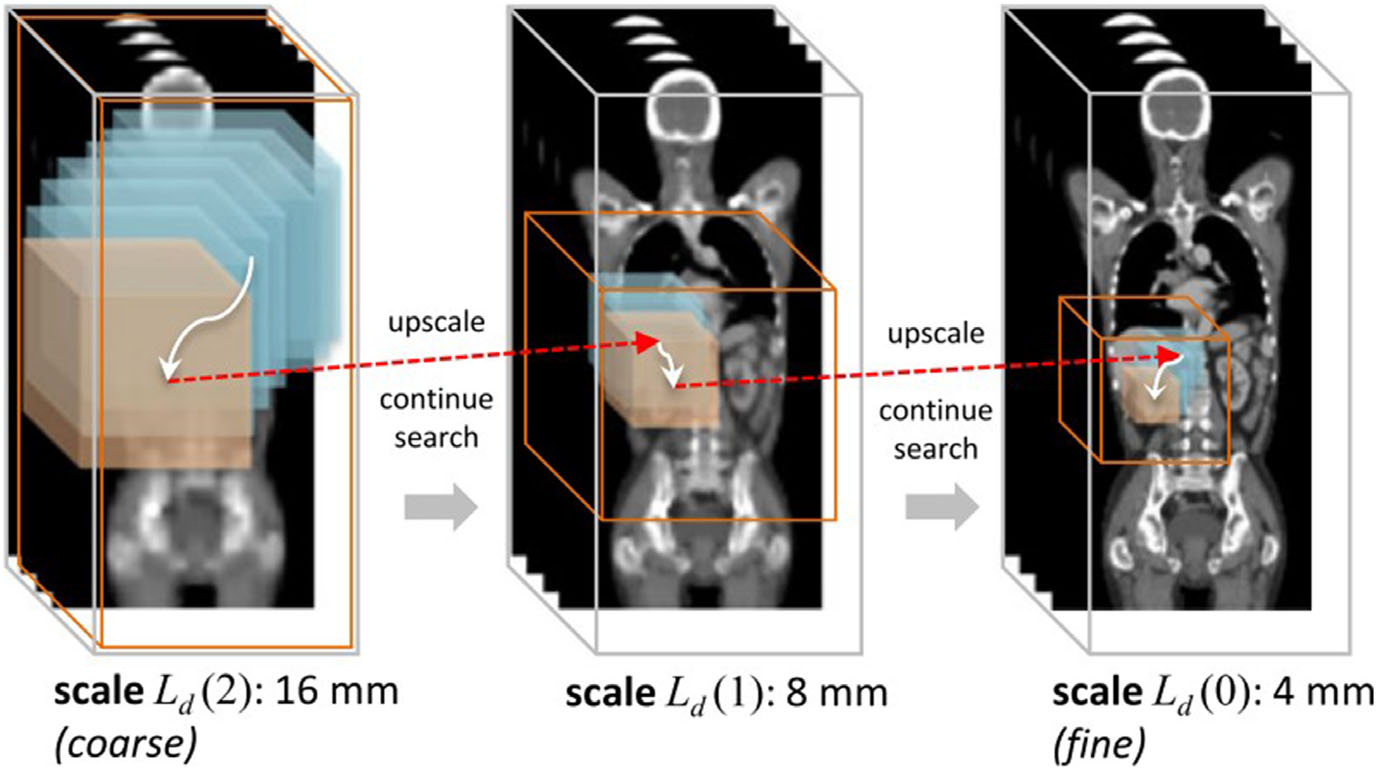
The trajectory of search the anatomical landmark across images of multiple scale‐levels. Courtesy of Ghesu et al.^20^

However, the approach mentioned above was still preliminary. One of the biggest disadvantages was that it could not fully use the information at different scale. So, a multi‐scale deep reinforcement learning method was later proposed in.^20^ The search for the landmark started from the coarsest scale. Once the search was convergent, the work continued with progressively finer scales until the search met the finest scale's convergence criteria. Figure 11 illustrates this non‐trivial search process. $L_{d}$ is the scale level in the continuous scale‐space L, which can be calculated as:

(2) $$L_{d}\left(t\right)=\psi_{\rho }\left(\sigma \left(t-1\right)*L_{d}\left(t-1\right)\right)$$

$\psi_{\rho }$ is the signal operator and σ is the Gaussian‐like smoothing function.

Alansary et al.^22^ extended the work of Ghesu et al. by evaluating different types of RL agents. They compared the detection results DQN, double DQN (DDQN), dual DQN, and dual double DQN (dual DDQN) on three different modalities’ datasets, which included fetal US, cardiac MRI, and brain MRI.

Rather than detecting a landmark per agent separately, attempts were made by^23^ to detect multiple landmarks with multiple collaborative agents. With the assumption that the anatomical landmarks have implicit correlations with each other, the detection of one landmark could indicate the location of some other landmarks. For the action function approximator in this paper, the collaborative DQN (Collab‐DQN) was proposed. The weights of the convolutional layers were shared by all the agents, while the fully connected layers for deciding the actions were trained separately per agent. Compared to the methods that trained agents for each landmark separately, this multi‐agent approach reduced detection error by 50% while also using a shorter training time.

Some other contributions to RL for anatomical landmark detection include estimating the uncertainty of reinforcement learning agent,^24^ reducing the needed time to reach landmarks by using a continuous action space,^25^ and localization of modality invariant landmarks.^26^

##### Lesion detection

Object detection, also called object extraction, is the process of determining the class labels and locations of target objects in images or videos. It was one of the primary tasks in medical image analysis.^27^ An exemplary detection result can be used as the basis for improving the performance of further tasks like segmentation.

The mainstream approaches for lesion detection still rely on time‐intensive exhaustive search methods and deep learning methods that require a large amount of labeled data. Facing the current challenges and inspired by similar problems in landmarks detection,^21^, ^28^ implemented a deep Q‐network (DQN) agent for active breast lesion detection. The states ware defined as current bounding box volumes of the 3D dynamic contrast‐enhanced MR images. The reinforcement learning agent was able to gradually learn the policy to choose between actions to move, scale the bounding box, and localize the breast lesion. Specifically, the action set consisted of nine actions that could translate the bounding box forward or backward along the x‐, y‐, and z‐axes, scale up or scale down the bounding box, and trigger the terminal state. To further evaluate the effectiveness of applying reinforcement learning on lesion detection with limited data,^29^, ^30^ used a DQN as the agent to localize brain tumors with very limited training data. Unlike,^28^ their brain MR data comprised 2D slices. The environment was defined as the 2D slices overlaid with gaze plots viewed by the radiologist. Instead of using the bounding box, the states were the gaze plots the agent located. Three actions—moving anterograde, not moving, moving retrograde—helped the agent transfer to the next state. A positive reward was given if the agent moved toward the lesion and a negative reward was given if the agent moved away from the lesion. If the agent stayed still within the lesion area, it received a large positive reward or a large negative reward if outside of the lesion area. The experimental results showed that reinforcement learning models could work as robust lesion detectors with limited training data, reduce time consumption, and provide some interpretability.

Addressing the lack of labeled training data,^31^ exploited visual attention mechanisms to learn from a combination of weakly labeled images that only had class labels and a limited number of fully annotated X‐ray images. This paper proposed convolutional networks with attention feedback (CONAF) architecture and a recurrent attention model with annotation feedback (RAMAF) architecture. The RAMAF model can only observe one part of the image, which was defined as a state at a glimpse. The reinforcement learning agent needed to learn the policy to take a sequence of glimpses and quickly locate the lesion site. Each glimpse consisted of two image patches that shared the same central point, and the length of the glimpse sequence was fixed to be 7. The rewards were decided according to correct classification of the image and the labeled bounding box containing the central point. RAMAF detected 82% of overall bounding boxes with a much faster detection speed than other state‐of‐the‐art methods.

In addition to detecting lesions in static medical images (2D or 3D), the RL‐based system could also continuously track the lesions frame by frame.^32^ proposed a robust RL‐based framework to detect and track plaque in Intravascular Optical Coherence Tomography (IVOCT) images. Despite the pollution problem of speckle‐ noise, blurred plaque edges, and diverse intravascular morphology, the proposed method achieved accurate tracking and could be applied broadly.

Three different modules were included in the proposed framework. The features were extracted and encoded by the encoding feature module. Then, the information of scale and location of the lesion was provided by the localization and identification module. Another function of this module was to prevent overtracking. The most important module was the spatial‐temporal correlation RL module. Nine different actions were possible, including eight transformation actions and one stop action. The state S was defined as three tuples: S=(E,HL,HA). Here, the E represented the encoded output features from the FC1 layer, HL was the collection of recent locations and scales, and HA represented the recent ten sets of actions. 8000 IVOCT images were used to evaluate the framework. With a strict standard (IOU > 0.9), the RL module could improve the performance of plaque tracking on both the frame‐level and plaque‐level.

##### Organ/anatomical structure detection

In addition to detecting lesions, reinforcement learning can also be applied in organ detection.^33^ designed a deep Q‐learning agent to locate various organs in 3D CT scans. The state was defined as voxel values within the current 3D bounding box. Eleven actions, including six translation actions, two zooming actions, and three scaling actions, made sure that the bounding box can move to any part of the 3D scan. The agent was rewarded if an action improves the intersection over union score (IOU). Seventy scans were used for training and 20 scans were used for testing on seven different organs, including the pancreas, spleen, liver, lung (left and right), and kidney (left and right). This proposed method achieved a much faster speed than the region proposal and exhaustive search methods and led to an overall IOU score of 0.63.

Zang et al.^34^ managed to detect and segment the vertebral body (VB) simultaneously. The sequence correlation of the VB was learned by a soft actor‐critic (SAC) RL agent to reduce the background interference. The proposed framework consisted of three modules: Sequential Conditional Reinforcement Learning network (SCRL), FC‐ ResNet, and Y‐net. The SCRL learned the correlation and gave the attention region. The FC‐ResNet extracted the low‐level and high‐level features to determine a more precise bounding box according to the attention region. At the same time, the segmentation result was provided by the Y‐net. The state of the RL agent was determined by a combination of the image patch, feature map, and region mask and the reward was designed according to the change of attention‐focusing accuracy to elicit the agent to achieve a better detection performance. This proposed approach accomplished an average of 92.3% for IOU on VB detection and an average of 91.4% for Dice on VB segmentation.

The research of^35^ was the first attempt to use the multi‐agent RL in prostate detection. Two DQN agents located the lower‐left and upper‐right corners of the bounding box while sharing knowledge according to the communication protocol (Foerster et al., 2016). The final location of the prostate was searched with a coarse‐to‐fine strategy to speed up the search process and improve the detection accuracy. In more detail, the agents first searched on the coarsest scale to draw a big bounding box and gradually moved to a finer scale to generate a smaller and more accurate bounding box to detect the prostate. Compared to the single‐agent strategy (63.15%), this multi‐agent framework achieved a better average score of 80.07% for IOU.

#### 3.1.2 Assessment

Detection is a type of problem that is straightforward to formulate as a control or pathfinding problem. The states are defined as the pixel values that the agents observe at the current step, and the actions are defined as movements along the different axes of the environment plus some scaling factors. The simplicity of defining detections as RL problems is why agent‐based detection has the largest number of papers among all RL‐related image detection tasks. Though related work in this field is still growing, some challenges exist. The generalizability and reproducibility of the agent‐based methods still need further investigation. In practical applications, the quality and local features of the image may vary by the noise and distortion introduced in the imaging process. The trained agent may not always be capable of finding the target in clinical settings. Furthermore, the trigger of the termination state in the inference stage needs to be improved. The most used criterion is the presence of oscillation. However, adherence to this criterion may lead to very ineffective convergence, and the agent might even become trapped at a local optimum and never reach the global optimum. Real‐time detection is another direction that has caused more interest in recent years. RL has proven its fast detection capability through its non‐exhaustive searching strategy. However, in some high dimensional data, such as 4D images (3D plus temporal), the real‐time detection and tracking still need more investigation. Finally, the training process of the RL system, especially the multi‐agent system, is very time‐consuming, which may take days or even weeks to train on platforms with exceptional hardware. The hyperparameter‐tuning is also highly reliant on the designer's experience. A summary of the works we reviewed in this section is given in Table 1.

**TABLE 1:** Overview of RL in medical image object and lesion detection

Author	ROI	Modality	Algorithm	State	Action number	Reward design
^32^	Heart	OCT	ADNet	Spatial‐temporal locations correlation information	9	Change of intersection‐over‐union (IOU) index
^28^	Breast	MR	DQN	Current bounding volume	9	Change of intersection‐over‐union (IOU) index
^33^	Lung, Kidney, Liver, Spleen, Pancreas	CT	DQN	Voxel value of the current bounding box	11	Change of intersection‐over‐union (IOU) index
^31^	Lung	Xray	REINFORCE	The part of the image observed by the glimpse	Number of image pixels	Correctness of the classification; Location of the glimpse
^34^	Vertebral Body	MR	SAC	Combination of the image patch, feature map and region mask.	4	Change of attention‐focusing accuracy
^29^	Brain	MR	DQN	The gaze plots the agent locate	3	Whether moving towards the gaze plot that include the tumor
^35^	Prostate	MR	DQN	Voxel values contained in the bounding box	4	Change of the Distance and IOU between the bounding box and the target

*Indicate that the missing part is not clearly defined in the original paper.

### 3.2 Medical image segmentation

#### 3.2.1 Overview of works

##### Threshold determination

Sahba et al.^36^ first attempted to use RL for medical image segmentation. The key idea was to formulate this segmentation task as a control task with a simple Q‐learning agent that decides the optimal local thresholds and the post‐processing parameters. The quality of the segmentation was considered when designing the state. The segmentation threshold and size of the structuring elements were changed by taking a series of actions. Although it was preliminary research, the segmentation quality was both acceptable and significantly reduced the required human intervention when compared to the mainstream methods at the time, like active contour.

##### Pre‐locate the segmentation region

**FIGURE 12:**
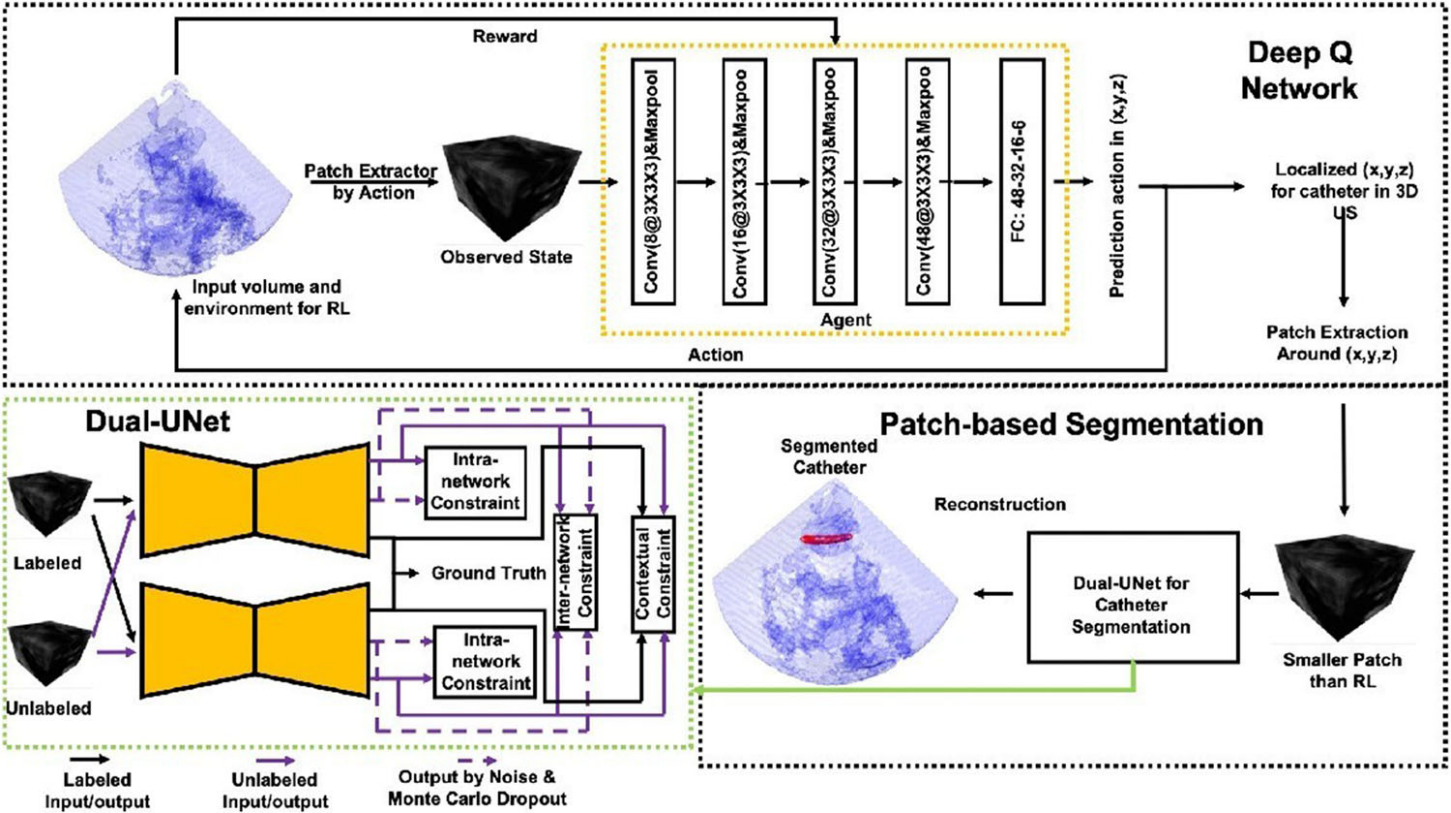
The semi‐supervised DQN‐driven catheter segmentation framework. Courtesy of Yang et al.^37^

Most supervised learning‐based catheter segmentation methods require a large amount of well‐annotated data. Yang et al.^37^ proposed a semi‐supervised pipeline, shown in Figure 12, that first used a DQN agent to allocate the coarse location of the catheter and then conducted patch‐based segmentation using Dual‐UNet. The RL agent reduced the need for voxel‐level annotation in the pre‐allocation stage. The semi‐supervised Dual‐UNet exploited unlabeled images according to prediction hybrid constraints, which improved the segmentation performance. The states were defined as the 3D observation patches, and the agent could update the states by moving the patch center point (*x*, *y*, *z*) along the *x*‐, *y*‐, and *z*‐axes of the observation space. Like the landmark detection problems, the agent gave a negative reward if the patch moved away from the target, a positive reward if the patch moved toward the target, and no reward if it stood still. Compared to the state‐of‐the‐art methods, this proposed pipeline required much less computation time and achieved an improved Dice score of at least 4% for segmentation performance.

##### Hyperparameters optimization

Instead of being directly involved in the segmentation process, RL agents can be applied to optimize the existing medical image segmentation pipelines.^38^, ^39^, ^40^ Bae et al.^38^ used RL as the controller to automate the searching process of optimal neural architecture. The required search time and computation power were significantly reduced by sharing the parameters while adopting a macro search strategy. Tested on the medical segmentation decathlon challenge, the authors asserted that this optimized architecture outperformed the most advanced manually searched architectures.

Qin et al.^39^ implemented an automated end‐to‐end augmentation pipeline using Dual DQN (DDQN) agent to reduce the effect of randomly augmented images harming final segmentation performance. By conducting trials and saving the experiences, the agent learned to determine the augmentation operations that were beneficial to the segmentation performance according to the feedback Dice ratio. Twelve different basic actions were used to change the state to achieve augmentation. The state was defined as the feature extracted from U‐Net. Horizontal flipping and cropping were found to be two of the most useful operations.

Yang et al.^40^ from NVIDIA integrated the highlights mentioned in the two previously reviewed papers. With an RNN‐based controller, this research automated the design process of hyperparameters and image augmentation to explore the maximum potential of the state‐of‐the‐art models. The optimal policy was learned using the proximal policy optimization to decide the training parameters. When tested on the medical decathlon challenge tasks, the RL‐searched model and augmentation parameters showed remarkable effectiveness and efficiency.

##### Segmentation as a dynamic process

After observing that many existing automated segmentation pipelines may often fail in real clinical applications,^41^ implemented multi‐agent reinforcement learning to interact with the users that could achieve iteratively refined segmentation performance. This multi‐agent strategy captured the dependence of the refinement steps and emphasized the uncertainty of binary segmentation results in design of states. Instead of defining the state as the binary segmentation result, it was formulated as $s_{i}^{\left(t\right)}=\left[b_{i},p_{i}^{\left(t\right)},h_{+,i}^{\left(t\right)},h_{-,i}^{\left(t\right)}\right]$, where *b*, *p*, and *h* are the value, previous prediction probability, and hint maps of voxel *i*, and *t* indicates the current step. The actions changed the segmentation probability by an amount a∈A, where *A* is the action set. Furthermore, the voxel‐wise reward was defined as $r_{i}^{\left(t\right)}=\chi_{i}^{\left(t-1\right)}-\chi_{i}^{\left(t\right)}\;$to refine the segmentation more efficiently, where χ was the cross entropy between the label $y_{i}$ and the segmentation probability $p_{i}$. The refined final segmentation result outperformed Min‐Cut,^42^ DeepGeoS(R‐Net),^43^ and InterCNN^44^ on all the BRATS20015, MM‐WHS, and NCI‐ICBI 2013 datasets. Though they published earlier than^41^ and adopted an older RL method to learn the policy,^45^ incorporated not only the user's background knowledge but also their intentions. The proposed framework follows a “Show‐Learn‐Act” workflow, which reduces the required interactions while achieving context‐specific and user‐specific segmentation.

#### 3.2.2 Assessment

Tackling image segmentation problems using RL agents provides us with an effective way to further optimize existing state‐of‐the‐art pipelines,^46^, ^47^, ^48^, ^49^ overcome a limited amount of training data, and interact with users to incorporate prior knowledge. Despite the novel of these methods, limitations still exist. The various definitions of states and actions may significantly influence the precision of the segmentation. In most works, the states are updated by a series of finite discrete actions to determine the final segmentation contours. Another problem is that the state design makes the agent only observe local or global information at any step. It would be interesting to see some methods in the future that can enable the agent to be capable of observing these two pieces of information at the same time. A summary of the works we reviewed in this section is given in Table 2.

**TABLE 2:** Overview of RL in medical image synthesis

Author	ROI	Modality	Algorithm	State	Action number	Reward design
^50^	Lung	CT	DQN	Control points coefficient sequences	*	Classification results of the pretrained classifier
^51^	Liver	MR	Pix‐GRL (AC‐based)	Pixel values of current image	3	Improvement of each pixel (pixel level); Improvement of pixels and surrounding pixels (region ‐level)
^52^	Cervix, Lymph node	Histopathology	PPO	Synthetic images	2	Validation classification accuracy
^53^	Cervix	Dose Volume Histogram (DVH)	Q‐learning	Current DVH weights	4 × 5	Change of the plan quality
^54^	Prostate	Dose Volume Histogram (DVH)	Q‐learning	Current DVH weights	5 × 5	Change of the plan quality

*Indicate that the missing part is not clearly defined in the original paper.

### 3.3 Medical image classification

Classification is one of the most basic tasks in medical image analysis. Common medical image classification tasks include disease diagnosis and prognosis, anomaly detection, and survivorship prediction. A label among the predefined classes would be assigned according to the extracted information from the image. Since most of the classification methods are fully supervised, these methods may often fail in real clinical settings due to a lack of high‐quality labeled data.

#### 3.3.1 Overview of works

##### Train with limited annotation

**FIGURE 13:**
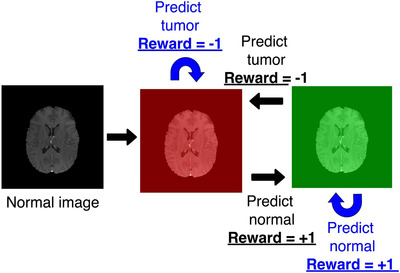
State transition and reward of a normal brain image. Courtesy of Stember & Shalu.^30^

To overcome this limitation, Stember & Shalu published two papers^30^, ^55^ which used a DQN agent in tandem with a TD model for accurate image classification with a minimal training set. The main workflows of these two papers were identical, except that the labels in the second paper were extracted from clinical reports using an SBERT.^56^ By overlaying the images with red or green masks, they formulated the classification problem as a behavioral problem, shown in Figure 13. The states were defined as the original greyscale image overlaid in green or red, where the red mask indicated a wrong prediction and the green mask indicated the correct prediction. The binary action (0 or 1) predicted the label of the image as normal (0) or tumor‐containing (1). If the prediction were correct, a +1 reward would be given to the agent; otherwise, a ‐1 reward would be given as a penalty. Compared with the supervised learning model trained on the same minimum dataset, the RL showed excellent overfitting resistance and high classification accuracy.

Another popular method to solve the lack of annotated data is to generate synthetic data. However, most medical synthesis pipelines do not assess the influence of the quality of these synthetic images in downstream tasks. Some misleading information in these synthetic data may skew the data distribution and thus harm the performance of the following tasks. To address this problem,^52^ designed a PPO RL controller that could select synthetic images generated by HistoGAN which adopted histogram‐based method for controlling generated image's color. Considering the potential relationship between the generated and existing data, they used a transformer to output the action decision with the features extracted by ResNet34 as input. The reward was designed according to the maximum validation accuracy in the last five epochs. Comparing the traditional augmentation, GAN augmentation, triplet loss metric learning, and centroid‐distance‐based selective augmentation, this Transformer‐PPO‐based RL selective augmentation achieved the best overall performance in the given classification task with an AUC score as high as 0.89.

##### Optimal sample/weight/ROI selection

In a clinical setting, it is common for radiologists to have obtained a considerable number images, but the annotation process might be time‐consuming and labor‐ intensive. A common approach to alleviate this problem is using active learning methods to select the most informative samples for annotations to improve the following tasks’ performance. Jingwen Wang et al.^57^ formulated active learning for medical image classification as an automated dynamic Markov decision process. The current state consisted of all the predicted values of the unlabeled images. This state then was updated by continuous actions to decide the unlabeled ones for annotations according to the optimal policy. The model was trained according to the deep deterministic policy gradient algorithm (DDPG), which consisted of an actor and a critic. A novice reward was designed to encourage the actor to focus on those samples that were incorrectly classified. Compared to other selective strategies, this RL framework achieved the best F1 score with all different percentages of training samples and a remarkable 0.70 score with only 40% labeled training data needed.^58^ combined meta‐learning and deep reinforcement learning for selective labeling. A bidirectional LSTM (BiLSTM) was used as the selector, and a non‐parametric classifier was used as the predictor. Instead of using the RL agent as a controller, this work used the policy‐gradient algorithm to optimize the objective function of the controller.

**FIGURE 14:**
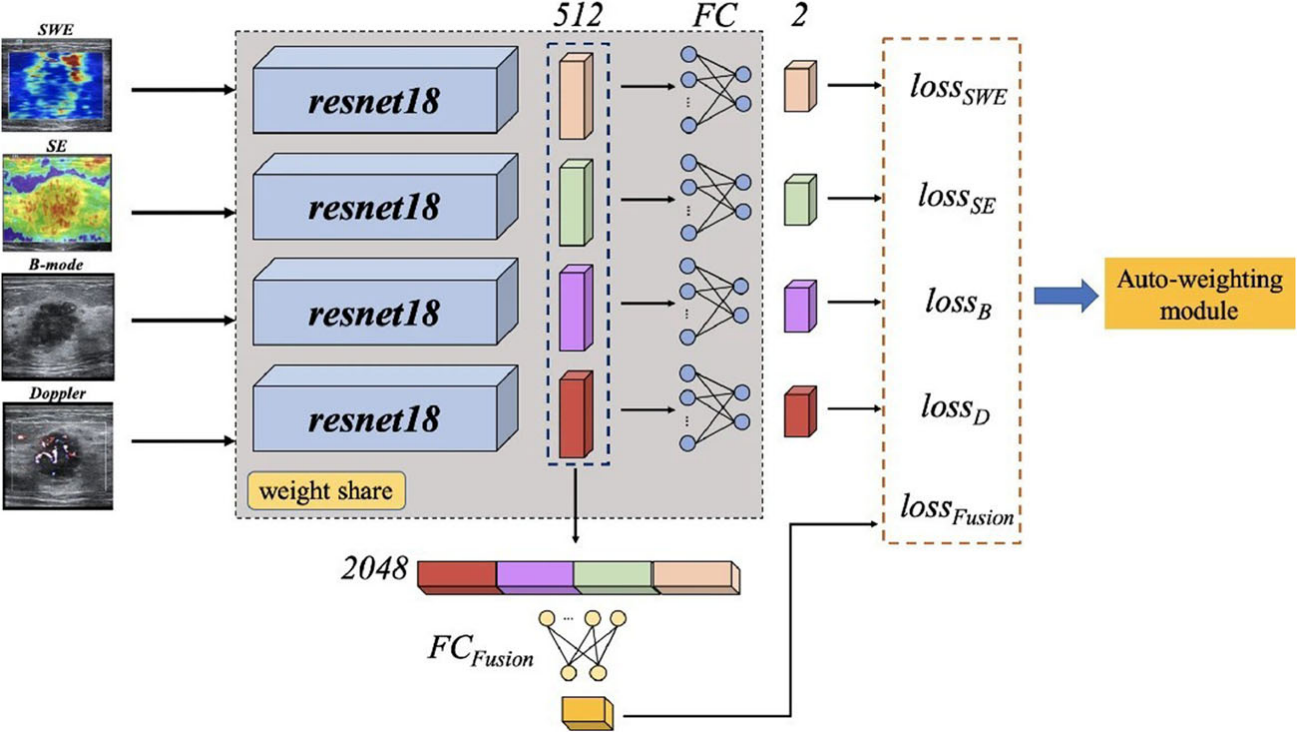
Schematic of the proposed multi‐modal model. The weights of the losses are determined by the RL module. Courtesy of Jian Wang et al.^57^

In clinical practice, an experienced physician usually makes diagnostic decisions from a combination of multi‐modal images. Similarly, many state‐of‐the‐art methods attempt to extract and integrate the information from various modalities to improve prediction performance. However, the weights of different modalities in this combination are hard to determine. Jian Wang et al.^59^ automated this as an end‐to‐end process controlled by a REINFORCE RL agent, as shown in Figure 14. There were four US modalities involved in this pipeline: B‐mode, Doppler, SE, and SWE. The model parameters were updated according to the global loss, which was a weighted summation of loss from each of the four modalities and a fusion loss calculated from the concatenated features. The state (weights) were updated (−0.2 or +0.2 or 0) at each step. Compared with other advanced single‐modality and multi‐modality methods on breast ultrasound datasets, this auto‐weighted RL method achieved the best overall performance with an accuracy as high as 95.43%.

For some types of medical images, like histopathology images, the resolution can be extremely high. Even though we have sufficient labeled data, it is still hard to perform the classification tasks due to the high computational intensity. Holding the belief that not all parts of the original images include valuable information (Xu et al., 2020), proposed an RL‐based pipeline for automated lesion region selection. Unlike the hard‐attention approaches, this RL selective attention method was end‐to‐end and fully automated, which included two major stages, the selection stage and the classification stage. The backbone of the selection stage was a recurrent LSTM that output a binary action that decided whether a cropped patch was useful for classification. In the classification stage, a soft‐attention network was used as the classifier. The reward that was fed back to the selector consisted of two‐parts, the training process reward, which represented the training stage accuracy, and the convergence reward, which represented the convergence performance. This selective strategy reduced the computation time by 50% and took less than 6 ms to infer a single image.

##### User interaction

Like the cases we reviewed in the segmentation section, well‐designed interactions with users can also improve the performance of classification tasks. However, instead of targeting the physicians as users,^60^ designed an RL controlled QA system that interacts with the patients. A CNN was pre‐trained to output a vector of the probabilities of the skin conditions. This vector was then concatenated with the vector of the patient's history answers about the symptoms, where each binary value in the vector represented the presence of a symptom. The action was designed to decide the next question to ask. The goal of the DQN agent was to maximize the possibility of classifying the correct condition when asking a specific question. Compared to CNN‐only and decision‐tree‐based approaches, this RL‐based symptom checker improved the classification accuracy by up to 20% and 10%, respectively.

#### 3.3.2 Assessment

The nature of image classification makes it hard to define classification as a control problem. So, instead of directly defining agent‐based classification frameworks, most works attempted to use RL agents to optimize the existing classification models’ hyperparameters or the image preprocessing process. The only two papers that applied agents to the classification task itself came from the same authors, who asserted that the agent‐based classification method is superior to other methods on small training sets and still needs to be validated with more relevant studies. We look forward to seeing more research that can effectively design image classification as the control problem. A summary of the works we reviewed in this section is given in Table 3.

**TABLE 3:** Overview of RL in medical image segmentation

Author	ROI	Modality	Algorithm	State	Action number	Reward design
^38^	Brain, Heart, Prostate	MR	REINFORCE	Hyperparameters of searched architecture	*	Change of the Dice Score
^41^	Brain, Heart, Prostate	MR	A3C	Combination of the voxel value, previous segmentation probability and hint maps	6	Decreased amount of the cross entropy
^39^	Kidney	CT	DDQN	Extracted high‐level feature	12	Change of the Dice ratio
^36^	Prostate	Ultrasound	Q‐learning	Segmented Objects	6	Change of segmentation quality
^45^	Ventricle	MR	Policy‐based	Local image appearance, anatomical details	Continuous	Difference between the location given by model and user
^40^	Atrium, Lung, Pancreas, Spleen	CT, MR	PPO	Parameter values	*	Change of segmentation performance
^37^	Catheter	Ultrasound	DQN	3D patches	6	Change of distance to the target

*Indicate that the missing part is not clearly defined in the original paper.

### 3.4 Medical image synthesis

Medical image synthesis is the process of generating synthetic images that include artificial lesions or anatomical structures of a particular or multiple image modalities. There are typically two types of medical image synthesis^61^: (i) inter‐modality, which transforms the image of a specific modality to another modality, for example, CT to MRI; (ii)intra‐modality, which transforms the imaging protocol within the same modality, for example, T1 sequence MRI to T2 sequence, or generates new images according to existing same‐modality images. Nowadays, medical image synthesis algorithms are dominated by GAN,^62^ for their ability to produce synthetic images with large diversity. Numerous GANs with different frameworks and techniques, such as Bayesian Conditional GAN,^63^ progressive growing GAN,^64^ self‐attention module,^65^ and deep supervision,^66^ have been proposed and achieved state‐of‐the‐art performance in various medical image synthesis applications. For the comprehensiveness of our review, we expanded the concept of image synthesis while including the radiation dose map planning in this section.

#### 3.4.1 Overview of works

##### Semantic map generation

Krishna et al.^50^ successfully combined reinforcement learning and style transfer techniques to synthesize fine CT images from a small image dataset. There were two major steps in this pipeline. First, a DQN agent automatically generated the semantic maps of the lung CT images. Next, the B‐splines and PCA interpolation were adopted to interpolate the semantic masks, thus providing texture information. The generated images had high resolution and were realistic enough to be used in other image analysis tasks.

##### Pixel value alteration

By designing a novice pixel‐level graph RL method,^51^ generated gadolinium‐enhanced liver tumor images from non‐enhanced images, thus avoiding the injection of toxic contrast agents. It is worth noting that this was the first paper that used an RL agent for image synthesis and the first attempt at designing agents, actions, and rewards at the pixel level. The design of agents was based on the actor‐critic structure but also integrated the idea of graph CNN. This graph‐driven, context‐aware mechanism enabled the model to capture both the small local objects and global features. The reward function $r_{i}^{t}=r{\left(e\right)}_{i}^{t}+\lambda {\left(w\right)}_{i}^{t}$ considered both the pixel‐level (first item) and region‐level (second item) rewards to make the measurement more accurate. According to the current states and rewards, the pixel‐level agents determined the actions to increase, decrease, or keep the pixel intensity. Trained and tested on 24 375 images, the model outperformed the existing state‐of‐the‐art method.^63^

##### Synthetic sample selection

Instead of focusing on directly synthesizing medical images, RL can also be applied in synthetic image assessment and selection. Ye et al.^52^ selected HistoGAN‐generated synthetic images according to their reliability and informativity. The selection process was formulated as a model‐free, policy‐based RL process stabilized by the Proximal Policy Optimization (PPO). The binary action was the output of the transformer model to decide if a fake image should be selected or discarded. The reward was designed to impel better accuracy in the following classification task. Compared with the unselected case, image augmentation using the chosen synthetic images improved the classification accuracy by 8.1% on the cervical data and 2.3% on the lymph node data.

RL has been widely applied in radiotherapy plan optimization to determine the optimal adaption time,^67^ tune the machine parameters,^68^ and decide the beam orientation.^69^ However, to keep in line with the scope of our review, we will only discuss agent‐based dose map planning.

##### Dose plan generation

Shen et al.^53^ was among the first works that attempted to use the RL agent to optimize the dose‐volume‐histogram (DVH) in a step‐by‐step manner, which led to the final dose map. The states were defined as the current weights of the DVH, and five actions per weight updated the states. The reward was defined as the change in the dose plan's quality, according to the WTPN's guidance. Compared with humans, the RL agents led to an average improvement of 10.7% of the final dose plan quality in high dose‐rate brachytherapy.

In one of their followed works,^54^ they applied the same idea to the external beam radiotherapy for prostate cancer patients. An end‐to‐end virtual treatment network (VTPN) was built to generate the optimum dose plan. They used ten patients for training and 64 patients for testing. With this VTPN‐based treatment planning pipeline, the original ProKnown score increased to 10.93 from 6.07.

##### Assessment

Works that use RL agents for image synthesis are still scarce in the literature. There are three types of different strategies for the general meaning synthesis: semantic map generation, pixel‐level value alteration, and synthetic sample selection. Compared to other state‐of‐the‐art methods, the agent‐based approach does not perform significantly better and has time‐consuming training. Additionally, we observed that the authors of these agent‐based synthesis works did not continue to publish any related works, which suggested a decline in interest.

The main idea for the agent‐based dose map generation is to update the DVHs step by step. Though this seemed to improve the treatment plan quality, the reward function was not fully based on clinical criteria, and the plan quality was only measured according to the DVHs, which is too simple a criterion and can result in undeliverable plans. More works are encouraged to evaluate and improve this agent‐based method for more challenging treatment planning scenarios. A summary of the works we reviewed in this section is given in Table 4 .

**TABLE 4:** Overview of RL in medical image classification

Author	ROI	Modality	Algorithm	State	Action number	Reward design
^60^	Skin	Dermascope	DQN	Patient's history answers + output probability of pretrained CNN	300	Probability of correct condition if asked the question
^58^	Chest	Xray	Policy Gradient	*	*	*
^30^	Brain	MR	DQN + TD	Image overlaid in red or green	2	Classification correctness
^55^	Brain	MR	DQN + TD	Image overlaid in red or green	2	Classification correctness
^57^	Breast	Ultrasound	REINFORCE	Weights	3	Classification correctness
^57^	Chest	CT	DDPG	Predictions of the unlabeled images	Continuous	Possibility of being classified incorrectly
^52^	Cervix, Lymph Node	Histopathology	PPO	Selected images	2	Maximum validation accuracy of last epochs
^70^	Breast	Histopathology	Policy Gradient	Learning status representation + Incoming data statistics	2	Performance of the selection mechanism

*Indicate that the missing part is not clearly defined in the original paper.

A summary of the works we reviewed in this section is given in Table 5.

**TABLE 5:** Overview of RL in medical image registration

Author	ROI	Modality	Algorithm	State	Action number	Reward design
^75^	Nasopharynx	CT‐MR	A3C	3D tensor composed of the moving and the fixed image	8	Distance between the transformed landmark and the reference landmark
^74^	Prostate	MR‐MR	Q‐learning	Spatial transformation parameters	30	The reduction of distance between the current parameters to the ground truth parameters
^71^	Spine, Cardiac	CT‐CBCT	Q‐learning	The rigid‐body transformation matrix	12	The reduction of distance between the current transformation to the ground truth transformation
^84^	Chest, Abdomen	CT‐Depth Image	Dueling DQN	3D tensor including cropped image pair	6	Small constant during the exploration, and big constant at termination with sign determined by change of constant
^83^	Brain, Liver	MR‐MR, CT‐CT	SPAC	Pair of fixed images and moving image	*	The change of the dice score
^73^	Spine, Cardiac	CT‐CBCT	Dilated FCN	The rigid‐body transformation matrix	12	The reduction of distance between the current transformation to the ground truth transformation
^72^	Spine	CBCT‐Xray	Q‐learning	Transformations in SE(3)	12	Reduction of distance to the ground truth transformation
^85^	Nasopharynx	CT‐MR	A3C	Concatenation of the fixed and moving image	8	Distance between the transformed landmark and the reference landmark

*Indicate that the missing part is not clearly defined in the original paper.

### 3.5 Medical image registration

Image registration is the process of transforming two different images into the same coordinate system. In the medical imaging domain, the registration could be inter‐patient, intra‐patient (but at different time points), inter‐modality (e.g., MRI, CT, CBCT, Ultrasound), and inter‐dimensionality (2D‐3D). The transformation models may also vary depending on the properties of the registration pair. It can generally be categorized into rigid, affine, and deformable transformation, where the rigid transformation is the simplest method that used parametric models. Traditionally, the registration was completed via optimizing the similarity metrics. However, the high dimensionality of the medical images and the tissues’ deformations and artifacts made this method unstable or sometimes even unfeasible. The emergence of agent‐based methods tackled the registration problem from a different angle, the formulation of the MDP process. Controlled by RL agents, the registration tasks achieved unprecedented robustness and precision.

#### 3.5.1 Overview of works

##### Rigid registration

The first attempt to use an agent‐based method to solve the registration problems was by.^71^ Unlike the standard methods that focused on matching metrics optimization, this agent‐based approach attempted to find the optimum sequence of actions that could align the images for registration. To train the agent with limited data, they first obtained synthetic data by dealigning the labeled training pairs. The registration process was done in a two‐step hierarchical manner to ensure robustness and accuracy. A coarse registration was first done in a broader field of view (FOV) with lower resolution, followed by the fine alignment on the full resolution image. This proposed method outperformed the ITK registration method, Quasi‐global search, and semantic registration methods on both the spine and heart 3D‐3D registrations to a large extent. This work confirmed the feasibility of agent‐controlled registration.

Based on the ideas from^71^, ^72^ evaluated the performance of this pipeline for 2D‐2D and 3D‐3D intra‐modality registration. This chapter of the book can serve as an effective tutorial because they discussed the state, action space, and reward design in detail. In,^73^ they attempted to use a multi‐agent attention mechanism to solve the 2D‐3D registration for images with severe artifacts. Instead of choosing the commonly used CNNs, this work adopted dilated fully‐CNN (FCN) as the backbone of the agent. This strategy reduced the registration problem's degrees of freedom from eight to four, which significantly improved training efficiency.

Moreover, they implemented the auto‐attention mechanism to overcome the strong artifacts in the 2D images. This attention was achieved through the use of multiple local agents to learn and decide the actions, and only the agents whose confidence scores were above a certain threshold (0.67) were selected. There were six pairs of actions (negative and positive) of the Special Euclidean Group SE(3). The framework was tested on both CBCT and more complex surgical data. In the more complex scenario, the multi‐agent attention mechanism showed much better robustness, signified by less performance degradation, than the corresponding single‐agent system.

##### Non‐rigid registration

So far, we have tackled exclusively rigid problems. Krebs et al.^74^ first attempted non‐rigid registration with a limited number of real inter‐subject pairs. Both the inter‐ and intra‐subject pairs were used for training. The intra‐subject pairs were generated as an augmentation step to compensate for the insufficient number of inter‐subject pairs. The authors built statistical deformation models to serve as a low‐dimensional representation of the problem because non‐rigid registration has more degrees of freedom than rigid registration. The fuzzy action control both minimized the possible number of actions and guaranteed the robustness of the. Experiments were conducted on both the 2‐D and 3‐D MR prostate images with median Dice scores of 0.88 and 0.76.

##### Lookahead inference

To further improve the registration performance of rigid registration,^75^, ^76^ incorporated trained networks with the lookahead inference. More specifically, the long‐short‐term‐memory‐machine (LSTM), specialized in tackling sequential data, was used to extract the spatial‐temporal features. The fixed and moving images formed a 3D tensor to represent the state updated by transformations, including translations (+/− 1 pixel), rotations (+/−1 degree), and scaling (+/− 0.05). In the testing phase, to make sure that the agent could reach the terminal state, they adopted a Monte Carlo rollout strategy to simulate different searching trajectories. The final transformation matrix was calculated as the weighted average of matrices from all the trajectories. Compared with other regression‐based and agent‐based methods, the addition of the lookahead and Monte Carlo rollout mechanisms improved the robustness and accuracy of multimodal image registration tasks.

#### 3.5.2 Assessment

From the works we have reviewed, it is not hard to see that agent‐based registration methods have comparable or even better performance than the intensity/deep‐ similarity‐based methods. However, most of the papers can only solve the rigid‐registration problems. For the non‐rigid transformer, however, the high‐dimensional state‐space and large number of degrees of freedom may impede the agents from efficient convergence. So, researchers may have to transform the transformation space into a lower‐dimensional space before applying the RL agents for registration. Another problem is that the agent may inherit the inefficiency from some similarity metrics used as the loss function during the training process, so novice loss functions should be designed for the RL frameworks. Finally, the methods that directly predict the transformation are still developing quickly. Many state‐of‐the‐art papers are emerging, while only a few papers are looking into agent‐based registration, which shows the low interest of researchers in this field.

## 4 DISCUSSION

Among all of the methods reviewed in this paper, we believe some future directions might be more promising. Medical image detection is the type of task RL has been most widely applied to. It is natural to formulate the detection problems as control problems optimized by RL, with the current attention region as the state, moving a certain number of pixels along dimensions of the image as actions, and defining the distance between the current position and the target position as rewards. The most effective method for this type of problem might be using the multi‐agent framework to search for the target in a coarse‐to‐fine‐resolution manner. This method may achieve outstanding precision while significantly improving search efficiency to achieve real‐time tracking. For medical image segmentation, using RL agents to pre‐locate the segmentation region might be one of the most promising directions. Cropping of the image is one crucial preprocessing stage of image segmentation. So far, this process still relies on human annotation, which is a luxury and experience‐demanding. RL agents, however, can embed this process as part of the training pipeline and learn the best segmentation region to boost the performance of downstream segmentation tasks. Among all of the RL methods used for classification, what we find most interesting is RL‐enabled user interaction. During the training process, errors and biases may occur, so the inference from the human experts will provide extra information to help the models learn the most valuable features. This interaction can also proactively choose what unlabeled data to label next, thus reducing the number of annotations needed while achieving better classification performance. In the category of image synthesis, what provokes medical researchers most might be the synthetic sample selection. It is often hard to qualitatively evaluate the quality of synthesized images. The decision made by RL models guarantees the reliability of these samples, thus further securing the effectiveness of downstream tasks using these synthetic images. Using RL for non‐rigid registration may gain even more attention in the future. Compared to rigid registration, non‐rigid registration is especially hard due to its high dimensionality. RL surpasses this barrier by decomposing the one‐shot transformation prediction into step‐by‐step parameter optimization to iteratively refine image alignment.

### Model‐free vs. model‐based

The agent‐based frameworks we have reviewed all belong to the model‐free category. However, the model‐based algorithm is another important subclass of RL. One possible reason why researchers did not attempt to use model‐based RL is that it is hard to form the internal model of the environment because of the high dimensionality and large size of medical image data. However, the model‐based algorithms have higher sample efficiency than the model‐free algorithms. We are looking forward to future works on model‐based RL for medical image tasks with small‐scale labeled data.

#### 4.1 Challenges

Many challenges still impede medical imaging researchers from applying RL in their works. The long training times and intensive computational requirements are important considerations prior to starting RL‐related research. Though RL has proved its efficiency in the inference phase of many tasks, it often takes at least a few days longer, even on some of the cutting‐edge GPUs, to learn from numerous trials and errors.

As previously mentioned, the design of the RL problems can be tricky. A slightly different design of the state, action, or reward may lead to a totally different performance and some models may even fail to converge. The choice of hyperparameters of the RL frameworks also depends on the designers’ experience with low explainability. Researchers may take days to experiment to find desirable parameters.

The low stability and reproducibility are other primary concerns.^77^ Following the same workflows, some RL agents may fail to work as well as described in the original paper. The lack of reproducibility is further exacerbated when the input data source is changed. Finally, the scarcity of RL‐related works, especially those with public source codes, greatly limits one's ability to reproduce results found in the literature.

Additionally, there are more accessible state‐of‐the‐art methods that can solve some types of medical imaging problems, and RL did not show a significant advantage over them. For example, it is still mainstream to use GANs for medical image synthesis, and many outstanding papers are emerging each year, which has caused interest in agent‐based methods in these fields to fade away.

#### 4.2 Future perspectives

The field of RL has developed very quickly in recent years, and many new theories or strategies have been proposed. However, the applications of RL in medical image analysis did not keep pace with this expansion. Here, we summarized some improvements that may lead to future trends of agent‐based medical imaging.

##### 4.3.1 Hierarchical reinforcement learning

Hierarchical reinforcement learning (HRL) aims to improve the agent's efficiency when facing some complicated problems. The main idea is to disassemble the final task into several subtasks in a hierarchical structure. There are three major subclasses of this type of framework: (i) HRL based on spatiotemporal abstraction and intrinsic motivation^78^; (ii) HRL based on an internal option^79^; (iii) Deep successor RL. These methods can potentially improve the current agent‐based pipeline when solving some high‐dimensional 3D image data or even 4D tracking data.^80^

#### 4.3.2 Multitask and transfer reinforcement learning

For all reviewed works, the trained RL agent can only perform the specific task trained for. While transfer learning is prevalent in deep learning for medical image analysis, it is reasonable to consider reusing some pre‐trained RL agents for similar but different new tasks. Agent‐based transfer learning can be categorized as behavioral transfer and knowledge transfer.^81^ By implementing the transfer learning in the current RL frameworks, we no longer have to train the agent to learn the complete policy from scratch. This would significantly reduce the training time and improve the frameworks’ generalizability.

#### 4.3.3 Active reinforcement learning

Active learning has a two‐fold meaning here: (i) Interaction with the users to incorporate users' prior knowledge; (ii) The agent decides what data to be labeled and what data will be trained. This active learning strategy can help the agent to understand the users' intentions and get maximum performance with minimum annotated data. However, this requires the involvement of human users, physicians in our case, in the process, so it might not be easy to be implemented in practice.

## 5 CONCLUSIONS

In this work, we witnessed the success of some researchers’ works that effectively turned traditional image analysis tasks into RL‐style behavioral or control problems. The basic concepts of reinforcement learning were first summarized, and then a comprehensive analysis of applications of RL agents for different medical image analysis tasks was conducted in different sections. Under each section, the formulations of RL problems were discussed in detail from different angles. As the essential elements of the RL systems, the choices of algorithms, state, actions, and rewards are highlighted in the tables. In conclusion, our review paper offers a simple and clear way for medical researchers to understand RL's concept and importance, thus changing the nature of research in medical image analysis in multiple ways. By posing the traditional medical imaging problems as controls optimization, these RL‐based methods provide researchers with a way to tackle problems and create new paradigms for solving current obstacles, especially with limited data and computation power. Equipped with knowledge of RL, data scientists can further optimize their models, thus pushing the performance boundaries forward. More importantly, RL methods can often surpass the performance of human supervisors while providing researchers and scientists with new perspectives and deeper understandings of these medical image analysis challenges. We hope that readers can find commonalities from these works, further understand the principles of reinforcement learning, and apply reinforcement learning in their future research.

## AUTHOR CONTRIBUTIONS

**Mingzhe Hu**: Conceptualization, Methodology, Investigation, Writing—Original Draft, Visualization. **Jiahan Zhang**: Methodology, Writing—Review & Editing. **Luke Matkovic**: Writing—Review & Editing. **Tian Liu**: Writing—Review & Editing, Supervision. **Xiaofeng Yang**: Conceptualization, Methodology, Writing—Review & Editing, Supervision, Project administration, Funding acquisition.

## CONFLICT OF INTEREST

None.

## Data Availability

The authors are not aware of any affiliations, memberships, funding, or financial holds that might be perceived as affecting the objectivity of this review.
